# Evaluating Behavioral and Linguistic Changes During Drug Treatment for Depression Using Tweets in Spanish: Pairwise Comparison Study

**DOI:** 10.2196/20920

**Published:** 2020-12-18

**Authors:** Angela Leis, Francesco Ronzano, Miguel Angel Mayer, Laura I Furlong, Ferran Sanz

**Affiliations:** 1 Research Programme on Biomedical Informatics Hospital del Mar Medical Research Institute Department of Experimental and Health Sciences, Pompeu Fabra University Barcelona Spain

**Keywords:** depression, antidepressant drugs, serotonin uptake inhibitors, mental health, social media, infodemiology, data mining

## Abstract

**Background:**

Depressive disorders are the most common mental illnesses, and they constitute the leading cause of disability worldwide. Selective serotonin reuptake inhibitors (SSRIs) are the most commonly prescribed drugs for the treatment of depressive disorders. Some people share information about their experiences with antidepressants on social media platforms such as Twitter. Analysis of the messages posted by Twitter users under SSRI treatment can yield useful information on how these antidepressants affect users’ behavior.

**Objective:**

This study aims to compare the behavioral and linguistic characteristics of the tweets posted while users were likely to be under SSRI treatment, in comparison to the tweets posted by the same users when they were less likely to be taking this medication.

**Methods:**

In the first step, the timelines of Twitter users mentioning SSRI antidepressants in their tweets were selected using a list of 128 generic and brand names of SSRIs. In the second step, two datasets of tweets were created, the *in-treatment* dataset (made up of the tweets posted throughout the 30 days after mentioning an SSRI) and the *unknown-treatment* dataset (made up of tweets posted more than 90 days before or more than 90 days after any tweet mentioning an SSRI). For each user, the changes in behavioral and linguistic features between the tweets classified in these two datasets were analyzed. 186 users and their timelines with 668,842 tweets were finally included in the study.

**Results:**

The number of tweets generated per day by the users when they were in treatment was higher than it was when they were in the *unknown-treatment* period (*P=*.001). When the users were in treatment, the mean percentage of tweets posted during the daytime (from 8 AM to midnight) increased in comparison to the *unknown-treatment* period (*P*=.002). The number of characters and words per tweet was higher when the users were in treatment (*P=*.03 and *P*=.02, respectively). Regarding linguistic features, the percentage of pronouns that were first-person singular was higher when users were in treatment (*P=*.008).

**Conclusions:**

Behavioral and linguistic changes have been detected when users with depression are taking antidepressant medication. These features can provide interesting insights for monitoring the evolution of this disease, as well as offering additional information related to treatment adherence. This information may be especially useful in patients who are receiving long-term treatments such as people suffering from depression.

## Introduction

### Background

Depression is one of the most common mental disorders [1]. According to the World Health Organization, depression affects more than 322 million people of all ages globally, being a leading cause of disability worldwide [2]. The proportion of people with depression went up by around 18% between 2005 and 2015 [3]. This mental disorder constitutes a challenge for society and health care systems due to devastating personal and social consequences and the associated economic costs [4-13]. In spite of the high prevalence of depression and the efforts of health care services to improve its management, this health condition remains underdiagnosed and undertreated [14].

In the case of moderate and severe forms of depression, pharmacological treatment can improve the quality of life of these patients [4]. There are several types of antidepressant drugs, and among them, selective serotonin reuptake inhibitors (SSRIs) are currently the most prescribed antidepressants around the world. For instance, according to the Spanish Agency for Medicines and Health Products [15], SSRIs constitute more than 70% of all antidepressants prescribed in Spain. They have fewer side effects than other antidepressants [16], show a good risk-benefit ratio [17,18], are safer and better tolerated [19], and exhibit a reduced risk of toxicity in overdose in comparison to tricyclic antidepressants [20]. They are commonly used as first-line treatment for depression [21-23] and are usually prescribed as maintenance therapy to prevent relapse [4,23-26]. SSRIs include the following drugs: fluvoxamine, fluoxetine, paroxetine, sertraline, citalopram, and escitalopram [17].

Furthermore, although social media platforms have typically not been created with health-related purposes in mind [27,28], millions of people publicly share personal health information on social media platforms every day [29,30]. For this reason, these platforms represent an important source of health information that is faster and more broadly available than other sources of health information, being unsolicited, spontaneous, and up to date. Infodemiology approaches have been developed and applied to better understand the dynamics of these platforms when used as a health information source [31-33]. In this context, social media users share health-related information, such as experiences with prescribed drugs [34], cancer patients’ sentiments [35], opinions on vaccines [36], or online conversations on epidemic outbreaks [37]. The massive data from social media can be monitored and analyzed by using natural language processing and machine learning technologies, providing new possibilities to better understand users’ behavior [30], including automatic identification of early signs of mental disorders [38-40]. In particular, it is typical for people suffering from depression to talk about their illness and the drugs they are taking [41-43].

Twitter is a very popular microblogging platform with more than 330 million active users worldwide [44]. Tweets, freely available in almost 90% of users’ accounts, provide a huge amount of data that can collected in real time [28,30,33,45-48]. Twitter users post short messages about facts, feelings, and opinions, including about health conditions [49].

Mining of drug-related information from Twitter has been applied in the pharmacovigilance field [27,50]. Some pharmacovigilance studies carried out on Twitter studied specific cohorts by identifying users’ mentions of drug intake [37,51-53]. Other studies focused on adverse drug reactions, analyzing users’ tweets regarding adverse events and side effects associated with drug use, which were identified by means of generic or brand names [29,47,54,55]. In our previous study [49], we observed that Twitter users who are potentially suffering from depression show particular behavioral and linguistic features in their tweets. These features were related to an increase in their activity during the night, a different style of writing with increased use of the first-person singular pronoun, fewer characters in their tweets, an increase in the frequency of words related to sadness and disgust emotions, and more frequent presence of negation words and negative polarity. This information can be used as a complementary tool to detect signals of depression and for monitoring and supporting patients using Twitter.

### Objectives

In this paper, we aim to enrich our previous study [49] by focusing on analysis of the changes in behavioral and linguistic features of Twitter users in Spanish language, which may be associated with the antidepressant medication these users are taking. It is worth mentioning that users from Spanish-speaking countries are among the most active on Twitter in the world [56]. The study is focused on Twitter users who mention treatment with SSRIs, which are the most frequently prescribed antidepressants [15]. In particular, this study compares the characteristics of the tweets posted while users were probably taking SSRIs versus the tweets posted by the same users when they have a lower probability of taking this antidepressant medication. This analysis can contribute to better understanding how these drugs affect users’ mood. Although we found two additional studies describing changes in Twitter users’ language in some mental disorders [57,58], to the best of our knowledge, there are no other studies that analyze Twitter posts in Spanish language to detect behavioral and linguistic changes when the users are taking antidepressant medication.

## Methods

### Study Design

**Figure 1:**
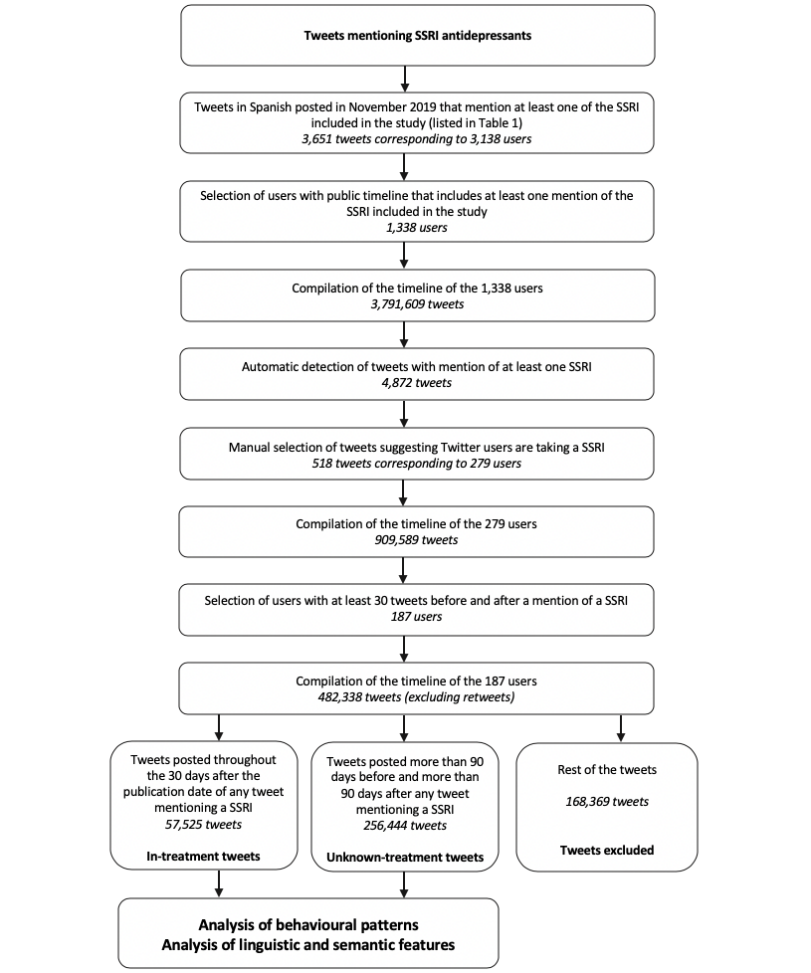
Flow diagram of the study process. SSRI: selective serotonin reuptake inhibitor.

This study was designed with the aim of analyzing the behavioral patterns and linguistic features of users who mention SSRIs in their Twitter timeline. The study was developed in several steps and focused on tweets written in Spanish. The flow diagram of the study is depicted in Figure 1.

As shown in Figure 1, two nonoverlapping datasets of tweets from users mentioning treatment with SSRIs were obtained: (1) The *in-treatment tweets dataset* was made up of the tweets posted throughout the 30 days after the publication date of any tweet mentioning SSRI intake. We assumed that these tweets were posted while the users had a high probability of being in treatment with an SSRI. (2) The *unknown-treatment tweets dataset* was made up of the tweets that were posted more than 90 days before or more than 90 days after the publication date of any tweet mentioning SSRI intake. We assumed that these tweets were posted while users had a lower probability of being in treatment with an SSRI than in the previous dataset.

These datasets were designed in a way that made it possible to carry out intrasubject comparisons, since the *in-treatment* tweets and *unknown-treatment* tweets datasets were obtained from the same Twitter users.

**Figure 2:**
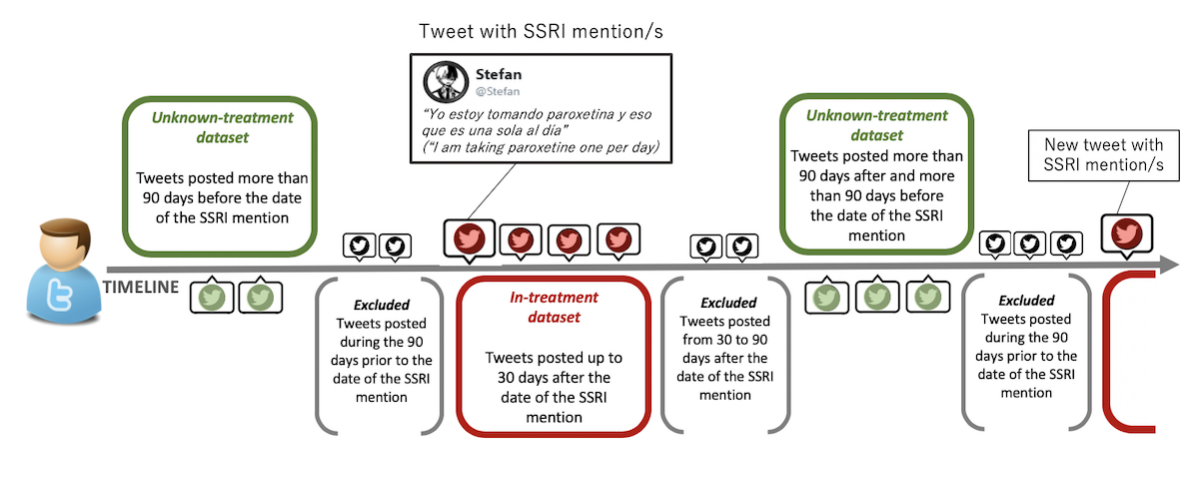
The in-treatment and unknown-treatment dataset selection strategy. SSRI: selective serotonin reuptake inhibitor.

The strategy for the selection of the tweets included in the two datasets is depicted in Figure 2.

### Data Collection and User Selection

The selection of the tweets and their users was based on the filtered real-time streaming support provided by the Twitter application programming interface [59]. In the first step, we selected tweets in Spanish that mention any of the SSRI generic and brand names used around the world. To obtain the generic and brand names, we performed searches on the following databases and resources: DrugBank [60], the Anatomical Therapeutic Chemical Classification System and the Defined Daily Dose of the World Health Organization [61], Wikipedia [62], and the Database for Pharmacoepidemiological Research in Primary Care [63]. The list of 135 generic and brand names obtained is shown in Table 1.

**Table 1:** Selective serotonin reuptake inhibitors (SSRIs) used in the study.

Generic name	Brand names
Fluvoxamina (fluvoxamine)	Dumirox, Faverin, Floxyfral, Fluvoxin, Luvox, Uvox
Fluoxetina (fluoxetine)	Prozac, Reneuron, Adofen, Luramon, Sarafem
Paroxetina (paroxetine)	Seroxat, Motivan, Frosinor, Praxil, Daparox, Xetin, Apo-oxpar, Appoxar, Aropax, Aroxat, Aroxat CR, Bectam, Benepax, Casbol, Cebrilin, Deroxat, Hemtrixil, Ixicrol, Loxamine, Meplar, Olane, Optipar, Oxetine, Pamax, ParadiseCR, Paradox, Paraxyle, Parexis, Paroxat, Paroxet, Paxan, Paxera, Paxil, Paxil CR, Pexot, Plasare, Pondera, Posivyl, Psicoasten, Rexetin, Seretran, Sereupin, Tiarix, Tamcere, Traviata, Xerenex, Xetroran
Sertralina (sertraline)	Aremis, Besitran, Zoloft, Altisben, Aserin, Altruline, Ariale, Asertral, Atenix, Eleval, Emergen, Dominium, Inosert, Irradial, Sedora, Serolux, Sertex
Citalopram (citalopram)	Seropram, Celexa, Akarin, C Pram S, Celapram, Celica, Ciazil, Cilate, Cilift, Cimal, Cipralex, Cipram, Cipramil, Cipraned, Cinapen, Ciprapine, Ciprotan, Citabax, Citaxin, Citalec, Citalex, Citalo, Citalopram, Citol, Citox, Citrol, Citta, Dalsan, Denyl, Elopram, Estar, Humorup, Humorap, Oropram, Opra, Pram, Pramcit, Procimax, Recital, Sepram, Szetalo, Talam, Temperax, Vodelax, Zentius, Zetalo, Cipratal, Zylotex
Escitalopram (escitalopram)	Cipralex, Diprex, Esertia, Essential, Heipram, Lexapro

The following 7 brand names of medicines have been excluded due to their semantic ambiguity: Essential, Motivan, Estar, Traviata, Pondera, Recital, and Emergen. These commercial names are, at the same time, very common words used with different meanings in Spanish, as we verified after reviewing a random sample of 200 tweets with mentions of these words. The number of tweets excluded because of their semantic ambiguity was 21,104. In the manual check of a random sample of 200 tweets, the mentions of SSRIs when using these words were 0% (0/200) in some cases, such as for Motivan and Estar, and 0.5% (1/200) for Recital. The final list of words included 128 generic and brand names of SSRIs.

Using the aforementioned 128 SSRI names, we collected 3651 tweets in Spanish posted during November 2019 with occurrences of the words listed in Table 1. These tweets were posted by 3138 different Twitter users and mentioned 33 different words from the list. The frequencies of these 32 words are shown in Table 2.

**Table 2:** Frequencies of SSRI names mentioned in Spanish tweets during November 2019.

SSRI mentions	Frequency
Prozac	998
Fluoxetina	756
Sertralina	542
Escitalopram	248
Citta	210
Citalo	109
Paroxetina	69
Pram	49
Fluvoxamina	40
Citalopram	33
Seroxat	22
Eleval	21
Lexapro	20
Opra	18
Casbol	14
Ariale	11
Zoloft	9
Altruline	9
Paxil	7
Akarin	7
Heipram	4
Aremis	4
Cimal	3
Tiarix	2
Seretran	2
Dominium	2
Citox	2
Atenix	2
Aserin	2
Talam	1
Dalsan	1
Celexa	1

In a second step, we crawled the public Twitter timelines of the 3138 users (until the 3200 most recent tweets for each user were retrieved). Given that retweets are not useful for analyzing the linguistic behavior of a particular user, the third step consisted of excluding the retweets and checking if the remaining tweets from each timeline included the mention of at least one SSRI. 1800 users were excluded by this filter, leaving a total of 1338 Twitter users. We obtained 3,791,609 tweets after compiling the timelines from these 1338 users. From these timelines, 4872 tweets mentioning at least one of the SSRIs from the list were automatically detected. These 4872 tweets were independently reviewed by two experts, a psychologist and a family physician, both with clinical experience. These experts manually selected the tweets that suggested that the user who posted the tweet was taking an SSRI on the date of posting. Examples of these tweets are shown in Textbox 1.

**Examples of tweets that positively or negatively suggest whether the user is taking an SSRI.**

Positive examples:

- “Eso de tener sueños raros debido a la fluoxetina se está saliendo de control.” (“Having odd dreams due to fluoxetine is getting out of control.”)
- “Yo tomo sertralina, como me lo receta el doctor y aún así a veces siento que el mundo donde estoy no es para mi. Ese susto esa angustia esas ganas de correr es algo que sólo el que lo padece lo entiende” (“I take sertraline as my doctor prescribes it to me and, even so, sometimes I feel that the world I’m living in is not for me. This fear this anxiety this desire to run out is something that only one who suffers from it can understand”)

Negative examples:

- “Ella debería tomar prozac, como Tic Tac” (“She should take prozac, like Tic Tac” [a candy brand])
- “La Paroxetina es un medicamento que pertenece a la familia de los antidepresivos inhibidores de la recaptación de la serotonina ¡Conoce más sobre él!” (“Paroxetine is a drug that belongs to the antidepressant family of serotonin reuptake inhibitors. Find out more about it!”)

The agreement between reviewers was 93.1% (4537/4872) with a Cohen kappa score of 0.68, indicating that there was substantial agreement between raters. The reviewers discussed and reached a consensus on the classification of the 335 tweets they classified differently. Finally, we obtained a total number of 518 tweets with one or more SSRI mentions, suggesting that the users who posted these tweets were taking an SSRI at the moment of posting. These tweets corresponded to 279 different users. Therefore, these users had two characteristics: first, the tweets on their timeline included at least one mention of SSRIs, and second, the text of tweets mentioning SSRIs suggested that the user was taking the antidepressant. In addition, we analyzed the tweets posted by each user that belonged to the two datasets (*in-treatment* and *unknown-treatment*; see Figure 1) by trying different minimum numbers of tweets per dataset (10, 30, 60, and 100 tweets) in order to include a user in the study. 10 tweets contained little information in terms of number of words or posting characteristics. In the cases of 60 and 100 tweets, the number of users included dropped dramatically. For this reason, we applied a requirement of a minimum of 30 tweets in both *in-treatment* and *unknown-treatment* datasets to keep the balance between the number of tweets and the number of users to be included in the study. After applying this requirement, 187 users were finally included in the study. The complete timelines of these users were compiled, totaling 668,842 tweets, which were reduced to 482,338 once retweets were removed. Out of these, 168,369 more tweets were excluded because they were posted on dates located outside the periods that qualified a tweet for being included in the *in-treatment* or the *unknown-treatment* datasets. Finally, 57,525 tweets were included in the *in-treatment* dataset and 256,444 in the *unknown-treatment* dataset.

### Data Analysis

The two datasets of tweets, *in-treatment* and *unknown-treatment*, were compared in order to determine the existence of behavioral and linguistic differences between the tweets generated by the users in each period. The features that were analyzed are listed in Table 3.

**Table 3:** Features of the tweets analyzed.

Features	Analyses performed
Distribution over time	Tweets per hour, tweets during daytime vs night, tweets per day, tweets during weekdays vs weekend
Length	Number of characters, number of words
Part-of-speech (POS)	Number of words by grammatical categories (part-of-speech tags)
Emotion analysis	Frequencies of emotion types
Negations	Frequencies of negation words
Polarity	Polarity of tweets on the basis of Spanish Sentiment Lexicon

Paired data statistical significance tests (paired *t* tests) were carried out whenever possible. The Benjamini-Hochberg false discovery rate was applied for multiple testing correction analysis [64]. The *P* values provided incorporate it.

The textual content of each tweet was analyzed using the same methodology and tools used in our previous study [49]. The textual content of each tweet was analyzed by means of the following steps: tokenization performed based on a customized Twitter tokenizer included in the Natural Language Toolkit [65]; part-of-speech (POS) tagging performed by means of the FreeLing Natural Language Processing tool in order to analyze the usage patterns of grammatical categories, such as verbs, nouns, pronouns, adverbs, and adjectives, in the text of tweets [66]; identification of negations performed by building upon a customized list of Spanish negation expressions, such as nada (nothing), nadie (nobody), no (no), nunca (never), and similar; identification of positive and negative words inside the text of each tweet using the Spanish Sentiment Lexicon [67]; and identification of words and expressions associated with emotions such as happiness, anger, fear, disgust, surprise, and sadness [68] by using the Spanish Emotion Lexicon [69].

The statistical analyses were carried out using Python 3.7, the Tweepy, SciPy, and Natural Language Toolkit libraries, and R version 3.6.2 (R Development Core Team), including the R “psych” package 1.9.12.31. All the aforementioned software tools are publicly available.

### Ethical Approval

The protocol used in this study was reviewed and approved by the Ethics Committee of Parc Salut Mar (approval number 2017/7234/1).

## Results

### Distribution Over Time

Several types of distribution-over-time analysis were performed in order to study the potential influence of being in *in-treatment* periods in comparison to *unknown-treatment* ones. The tweet hours were adjusted by the users’ time zone.

The mean duration of the time period analyzed of all the users was 28.2 months (SD 24.7); the mean of the total number of tweets analyzed was 307.6 (SD 336.0) for *in-treatment* periods and 1371.4 (SD 748.2) in the case of *unknown-treatment* periods. The mean number of tweets per day generated by users during *in-treatment* periods was 11.44 (SD 10.05); this number dropped to 9.07 (SD 7.21) in the *unknown-treatment* dataset with a mean difference of 2.37 (SD 9.72) between periods, which shows statistically significant differences between the two datasets (*t*_186_=3.33; *P<*.001).

The mean percentage of tweets posted during daytime (between 8 AM and midnight) was 64.30% (SD 14.83) when the users were *in-treatment* periods; this percentage fell to 61.78% (SD 13.69) during the *unknown-treatment* periods, with a mean percentage difference of 2.52% (SD 11.81), which implies statistically significant differences (*t*_186_=3.07; *P=*.004).

The mean number of tweets generated during the weekdays (from Monday to Friday) was 12.28 (SD 11.05) during *in-treatment* periods and 9.33 (SD 6.70) in the *unknown-treatment* periods, with a mean difference of 2.95 (SD 10.23) and statistically significant differences between the datasets (*t*_186_=3.93; *P<*.001). For the mean number of tweets generated during the weekends (Saturday and Sunday), it was 9.35 (SD 9.31) in the *in-treatment* period and 8.41 (SD 9.82) in the *unknown-treatment* period, with a mean difference of 0.94 (SD 10.92) that implies statistically significant differences between the datasets (*t*_186_=1.18; *P=*.23). The mean percentage of tweets posted on weekdays was 75.95% (SD 9.17) during *in-treatment* periods; the percentage went down to 74.40% (SD 5.31) in *unknown-treatment* periods, with a mean percentage difference of 1.56% (SD 8.9) that implies statistically significant differences between the two periods (*t*_186_=2.39; *P*=.02).

### Length

The average number of characters per tweet was 88.03 (SD 30.74) and 85.19 (SD 28.82) in the *in-treatment* and *unknown-treatment* datasets, respectively, with a mean difference of 2.84 (SD 17.70) and statistically significant differences between the periods (*t*_186_=2.19; *P*=.03). As for the number of words per tweet, the mean was 15.68 (SD 5.75) in the *in-treatment* dataset and 15.09 (SD 5.20) in the *unknown-treatment* dataset, with a mean difference of 0.59 (SD 3.54) and statistically significant differences (*t*_186_=2.28; *P*=.02).

### Links and Mentions to Other Users

The mean percentages of tweets that include at least one link were 23.10% (SD 16.16) and 23.27% (SD 15.29) in the *in-treatment* and *unknown-treatment* datasets, respectively, with a mean difference of −0.17 (SD 10.94), which is not statistically significant (*t*_186_=−0.23; *P*=.82).The mean percentages of tweets that include at least one mention of another Twitter user were 45.79% (SD 24.77) and 43.52% (SD 24.71) in the *in-treatment* and *unknown-treatment* datasets, respectively, with a mean difference of 2.27% (SD 12.13), which is statistically significant (*t*_186_=2.56; *P*=.01).

### Part-of-Speech

As for the analysis of the number of words by grammatical category (ie, part-of-speech) in each tweet, we also compared the *in-treatment* and *unknown-treatment* datasets. The mean percentage of words per grammatical category over the total number of words in each dataset is shown in Table 4. We considered the most relevant lexical POS such as verbs, nouns, pronouns, adverbs, and adjectives, excluding conjunctions, interjections, punctuations, determiners, adpositions, numbers, and dates.

Regarding the different types of pronouns, the mean percentages of personal pronouns in each dataset are shown and compared in Table 5.

**Table 4:** Percentages of part-of-speech words compared between in-treatment and unknown-treatment datasets.

POS^a^	*in-treatment* (%), mean	*unknown-treatment* (%), mean	Difference (%), mean (SD)	Paired *t* test	*P* value
Verbs	18.50	18.20	0.3 (1.28)	3.15	.002
Nouns	19.50	19.94	−0.44 (2.57)	−2.35	.02
Pronouns	9.19	8.93	0.26 (1.33)	2.61	.01
Adverbs	6.42	6.36	0.06 (0.84)	0.97	.34
Adjectives	6.05	6.21	−0.16 (0.95)	−2.34	.02

^a^POS: part-of-speech.

**Table 5:** Mean percentages of personal pronouns compared between in-treatment and unknown-treatment datasets.

Personal pronouns	*in-treatment* (%), mean	*unknown-treatment* (%), mean	Difference (%), mean (SD)	Paired *t* test	*P* value
1st person singular	49.50	47.80	1.7 (8.68)	2.67	.008
2nd person singular	14.77	16.07	−1.3 (6.17)	−2.88	.004
3rd person singular	22.13	22.86	−0.73 (5.79)	−1.72	.08
1st person plural	3.44	3.43	0.01 (3.43)	0.04	.96
2nd person plural	1.00	1.00	0 (1.22)	−0.01	.98
3rd person plural	5.60	5.39	0.21 (3.68)	0.77	.44

### Emotion Analysis

The mean percentages of the different emotions, obtained using the Spanish Sentiment Lexicon on the tweets posted in the two periods, are shown in Table 6.

**Table 6:** Mean percentages of different emotions compared between in-treatment and unknown-treatment datasets.

Emotion	*in-treatment* (%), mean	*unknown-treatment* (%), mean	Difference (%), mean (SD)	Paired *t* test	*P* value
Happiness	26.93	25.94	0.99 (5.82)	2.32	.02
Sadness	10.01	9.76	0.25 (4.20)	0.81	.41
Fear	3.20	3.02	0.18 (1.94)	1.23	.21
Anger	5.52	5.20	0.32 (2.71)	1.62	.11
Disgust	3.11	3.06	0.05 (1.97)	0.38	.69
Surprise	5.59	5.06	0.53 (2.42)	2.98	.003

### Negation Analysis

The mean percentages of tweets, among all users, that included one or more negation words were 27.66% (SD 10.54) and 26.59% (SD 9.87) for the *in-treatment* and *unknown-treatment* datasets, respectively, with a mean difference of 1.07% (SD 6.99), which is statistically significant (*t*_186_=2.10; *P=*.04).

### Polarity Analysis

As for the polarity of tweets, the percentage of tweets, among all users, with one or more positive words inside the text was 15.13% (SD 6.56) in the *in-treatment* dataset and 14.50% (SD 5.43) in the *unknown-treatment* dataset, with a mean percentage difference of 0.63% (SD 5.22; *t*_186_=1.66; *P=*.09). The percentage of tweets with one or more negative words was 7.97% (SD 4.40) in the *in-treatment* dataset and 7.54% (SD 3.52) in the *unknown-treatment* dataset, with a mean percentage difference of 0.43% (SD 3.58) (*t*_186_=1.64; *P=*.10). No statistically significant differences were detected in this analysis.

## Discussion

### Principal Findings

Social media platforms in general, and Twitter in particular, may provide useful information on how patients respond when they receive a pharmacological treatment, as has been shown in several studies in which social media has been used as a complementary source of pharmacovigilance and monitoring [34,70]. In this study, we analyzed the tweets of users who mentioned they were taking antidepressant drugs, in particular SSRIs, with the aim of detecting behavioral changes when they are more likely to be in treatment in comparison to periods in which they are less likely to be in treatment *(“in-treatment”* vs *“unknown-treatment”* periods).

The results of this study show that, in general, Twitter users significantly increased their activity of posting tweets during the *in-treatment* periods. This increase was more pronounced during weekdays than during weekends. We also observed a significantly greater proportion of tweets posted during the daytime during the *in-treatment* periods. These results are consistent with the results of our previous paper [49], in which we observed that the control group without signs of depression showed more tweet posting activity than the group of users with signs of depression, especially during the daytime and the weekdays. These results are also consistent with another paper that described the behavior in social media of people with self-reported depression [41], as well as with a study on the diurnal mood variation of patients suffering from major depressive disorder [71]. In summary, we can state that when considering tweet posting activity, the behavior of individuals suffering from depression becomes more similar to that of the general population when they are in treatment with SSRIs.

Likewise, the average number of characters and words per tweet were significantly higher when the Twitter users were in treatment with SSRIs, a finding that again points toward an increase in the activity of these treated users. In addition, the increase in the number of mentions per tweet can reflect a greater interest in interacting with other people. All these changes may be due to some improvement in their anhedonic symptoms because of the medication.

Regarding the linguistic analysis, we observed quantitatively slight changes between the *in-treatment* and the *unknown-treatment* periods, although in some cases they are statistically significant. These slight findings are not easily interpretable. In general, given that the style of writing of people suffering from depression is characterized by self-focus attention, which is associated with negative emotional states and psychological distancing in order to connect with others [72], we can conclude that when the studied subjects were in treatment, they improved some traits related to their posting activity as previously mentioned, but at the same time, their language maintained the features of people suffering from depression without a clear influence of the medication.

Emotion is another important aspect that characterizes people suffering from depression, and it was consequently analyzed. When the users were in treatment, they showed small but statistically significant increases in the happiness and surprise emotions, but not in sadness or other emotions (ie, anger, fear and disgust). As for the number of negations, the users slightly increased their use of these types of words during the *in-treatment* period. However, the polarity analysis did not show differences between the periods.

The increased activity observed on Twitter when the users were likely to be in treatment with SSRIs can be linked to improved emotional status in their happiness and surprise emotions. These changes are consistent with our previous observations on mood states of Twitter users without depression compared to those with depression [49]. However, the traits that are related to language, as indicated by the POS analysis and the use of negations, maintained a similar profile to that of subjects with depression, independently of the pharmacological treatment detected. These results denote that users with depression who are taking SSRIs show some mood improvements while receiving antidepressant treatment, but at the same time maintain an altered language pattern, which may be indicative of incomplete recovery.

On the basis of our statistically significant results, we may state that Twitter timelines can be used as a complementary tool to monitor subjects in order to detect adherence to treatment, which is an important problem in this kind of patient. Adherence to treatment is essential for disease remission [73-76]. According to some studies, it is common for patients suffering from depression to not maintain the duration of antidepressant treatment that is clinically recommended [4,18,77]. In summary, the follow-up of behavioral and language changes in users’ Twitter timelines can be useful for monitoring the evolution of depressive symptoms and the effect of treatments.

### Limitations and Future Directions

This type of study in general, and this one in particular, presents some limitations. For instance, we considered tweets written in Spanish and from public Twitter users’ timelines, and these users may be not representative of the general population or people suffering from depression [33,49,78,79]. Some studies have shown that Twitter users are often urban people with high levels of education, and they are generally younger than the general population [33,49,78,80,81]. We should also take into account that SSRIs are used in different types of depressive disorders and in other mental conditions. Moreover, we have no information about whether these drugs were taken in the context of a prescribed medical treatment or as a result of an inappropriate self-medication decision.

Another limitation may be the fact that Twitter users who share their personal drug intake may use words or expressions not included in the list of drug names employed in this study for streaming tweets, even though we tried to be exhaustive in the list of names used. Twitter texts are informal and limited by the number of characters, and they commonly include abbreviations, errors, or slang language [33,45]. All these issues can make it difficult to automatically extract drug mentions and link them to a formal lexicon [28,30,50,53,55]. Unlike clinical records that could be linked to domain resources, the lack of lay vocabularies related to health concepts and terminologies hinders the processing of social media texts [55]. In addition, the results obtained may depend on the particular drugs selected for the study [33], as well as on the periods of time set up for classifying the tweets into the *in-treatment* and *unknown-treatment* datasets. On the basis of the strategy applied for defining the groups of tweets to be compared (tweets generated just after mentions to SSRI intake vs tweets generated in periods far from any mention to the SSRI intake), there is some chance of misclassification; it is likely that not all the tweets in the first group were generated by users under actual SSRI treatment, and it is probable that some tweets of the second group have been generated by users under SSRI treatment.

Furthermore, we must take into account that data from social media posts contain irrelevant information. Although the proportion of useful information for the specific research purpose can be quite limited, it constitutes a useful starting point [28,30,51,53]. In this scenario, the human curation of tweets is a necessary step in this kind of analysis [34]. Even so, due to the different nuances that a tweet can involve, it is not easy to detect real drug intakes or firsthand experiences [24,46,52].

### Conclusions

Social media can be used to monitor the health status of people and, in particular, to detect symptoms or features related to diseases or health conditions by means of analysis of the users’ behavior and language on social media platforms. Moreover, the detection of changes in symptoms or other features when patients are taking medications can provide interesting insights for monitoring pharmacological treatments, as well as for following up on the evolution of the disease, detecting side effects, or providing information related to treatment adherence. Changes in some features, such as a decrease in activity on Twitter or of the length of tweets, an increase of self-focus through the use of the first-person singular pronoun, and changes in the happiness and surprise emotions could be used as complementary tools to detect the worsening of the psychological status of users suffering from depression, as well as to perceive lack of adherence to treatment. This information may be especially useful in patients suffering from chronic diseases who are receiving long-term treatments, as is the case for mental disorders in general and depression in particular. However, it is not possible to determine the specific reasons why individuals change their behavior and language on social media platforms in the framework of a disease and its treatment without performing a clinical assessment. Overall, this study shows the relevance of monitoring behavioral and linguistic changes in the tweets of persons taking antidepressants. These changes are likely to be influenced by the diverse stages of the disease and the therapeutic effects of the treatment that these Twitter users are receiving, opening a new line of research to better understand and follow up on depression through social media.

## Abbreviations

POS: part-of-speech
SSRIs: selective serotonin reuptake inhibitors
